# Predicting social isolation in maintenance hemodialysis patients using machine learning methods: a cross-sectional study

**DOI:** 10.3389/fpsyt.2026.1776298

**Published:** 2026-02-18

**Authors:** Ying Li, Wenwen Zhao, Boyang Wang

**Affiliations:** 1 College of Sports Science, Jishou University, Jishou, Hunan, China; 2 School of Nursing, Qilu Medical University, Zibo, Shandong, China; 3 Nephrology Department, Nephrology Department Shanghai East Hospital, Shanghai, China

**Keywords:** machine learning, maintenance hemodialysis, predictive modeling, random forest, risk stratification, social isolation

## Abstract

**Background:**

This study aims to develop and validate a machine learning-based risk prediction model for social isolation in maintenance hemodialysis (MHD) patients, and at the same time determine the key risk factors.

**Method:**

362 patients with MHD were recruited from a tertiary hospital in Shanghai and randomly divided into the training group and the detection group. We implemented and compared seven machine learning algorithms: Random Forest (RF), Decision Tree (DT), K-Nearest Neighbor (KNN), Logistic Regression (LR), Resilient Network (EN), Extreme Gradient Boosting (XGB), and Support Vector Machine (SVM).

**Result:**

In our MHD cohort, the incidence of social isolation was 45.856%. The comparative analysis shows that RF is the best prediction model (AUC = 0.95). Feature importance analysis identified significant predictors: Place of residence (1.277), Heart failure (HF) (0.559), Anxiety (0.306), Monthly household income (0.269), Age (0.255) Sleep condition (0.138).

**Conclusion:**

The prediction model based on RF has a good effect in identifying the social isolation risk of MHD patients. These findings enable clinicians to stratify high-risk populations and implement timely and targeted intervention measures, effectively reducing the risk of adverse consequences. Future multicenter studies should validate these results in larger cohorts.

## Introduction

Chronic kidney disease (CKD) has emerged as a significant global public health concern, with profound implications for healthcare systems worldwide (1). According to statistics, the global estimated prevalence of chronic kidney disease (CKD) is 13.4% (2). China has approximately 120 million patients with CKD, of which about 2 million have advanced to end-stage renal disease (ESRD) (3). For patients progressing to ESRD, maintenance hemodialysis (MHD) serves as the primary renal replacement therapy, playing a critical role in disease management. As dialysis duration extends, psychological disturbances stemming from treatment complications and comorbidities become increasingly prevalent. Social isolation represents a state of detachment from societal engagement, characterized by diminished participation in activities, reduced social interactions, and impaired interpersonal communication, often leading to adverse physical and psychological consequences (4). Among MHD patients, deteriorating physical function, increasing complications, and rising treatment costs collectively contribute to reduced social participation and functional decline, thereby elevating isolation risk. Studies have shown that the rate of social isolation among MHD patients ranges from 45.2% to 63.8% (5, 6). Growing evidence identifies social isolation as both a serious public health concern and a significant barrier to health-promoting behaviors (7). This condition is associated with multiple adverse outcomes, including physical function deterioration (8), elevated risks of dementia (9)and type 2 diabetes (10), psychological distress (e.g., loneliness and depression) (11), and increased mortality (12). Moreover, it negatively impacts both physical and mental health (8–12), restricts social engagement, and compromises quality of life (13). Conventional assessment methods demonstrate limited predictive accuracy y for this population, primarily due to the multifactorial etiology and substantial heterogeneity of contributing factors. These limitations largely reflect constraints inherent in traditional evaluation frameworks. Machine learning (ML) algorithms, however, offer distinct advantages by effectively processing diverse data types and scales, making them particularly suitable for developing patient-centered predictive models. This innovative approach holds substantial promise for optimizing treatment strategies and enhancing health monitoring in MHD populations.

ML algorithms are suitable for all types and sizes of data and have attracted great attention in developing patient-centered predictive/prognostic models (14). These models help optimize treatment plans and facilitate the monitoring and managing health conditions. In our research, we chose the commonly used machine learning models: logistic regression (LR), elastic network (EN), K-nearest neighbor (KNN), decision tree (DT), Extreme Gradient Boosting (XGB), support vector Machine (SVM), and Random Forest (RF). XGB employs a sophisticated boosting framework that systematically enhances model performance through several key mechanisms. The algorithm generates weak learners by optimizing a structured loss function, while incorporating advanced techniques such as pre-sorting and weighted quantiles to mitigate overfitting risks. These methodological innovations collectively contribute to superior generalization capacity, enabling robust predictive performance across diverse datasets (15). SVM is used to construct a model for limited data, so that the model can achieve the effect of minimizing structural risk (16). During the process of model construction, the best compromise position is sought between the complexity of the function and the accuracy of data analysis, so as to obtain the best generalization ability (17). DT is a classic classification method, which is an algorithm for summarizing classification rules from the training dataset (18). This method has relatively low requirements for the data. As it divides the independent variables one by one, it has achieved remarkable results in fields such as classification, prediction, and rule extraction at present (18). The EN model addresses overfitting through complexity regularization and enhances interpretability by jointly selecting relevant features (19). Using this model, highly accurate predictions can be made for the characteristics of unknown data. KNN is a type of classification algorithm. It can supervise the machine to perform calculations and use Markov distance, Euclidean distance, etc., to minimize the similarity, thereby determining the classification of a certain data to be measured (20). Logistic regression is used to convert the result of a certain continuous value obtained by linear regression into a probability value with an interval, and then handle classification problems based on the obtained probability value (21). RF is an algorithm capable of fusing multiple decision trees and belongs to an ensemble algorithm (22). The operation is simple. Whether in the training process or parameter adjustment, it can be carried out quickly. The RF estimation error is combined to evaluate the fitting and prediction accuracy of the combined tree learner (23, 24). Current research on ML for predicting social isolation risk has primarily focused on elderly populations and patients with schizophrenia or bipolar disorder (25–27), while attention to the broader population with MHD remains limited. Therefore, this study investigated the prevalence of social isolation in patients undergoing maintenance hemodialysis, aiming to assess the clinical applicability of machine learning models in predicting social isolation. Through comprehensive feature importance analysis, we further identified and validated the primary predictors of social isolation within this vulnerable population.

## Methods

### Methods study population and design

This prospective multicenter cohort study enrolled patients diagnosed with MHD at a tertiary hospital in Shanghai between January 2025 and June 2025. In accordance with the Declaration of Helsinki principles, written informed consent was obtained from all participants prior to study inclusion.

### Inclusion criteria

Diagnosis of MHD with a dialysis duration ≥6 months; Age ≥18 years; Clear consciousness; and Willingness to provide written informed consent.

### Exclusion criteria

Severe comorbidities (cardiac, cerebral, or other major organ system dysfunction); Language barriers or cognitive impairments; Documented psychiatric disorders or intellectual disabilities.

### General information and questionnaire data

#### Demographic characteristics

Our study comprised two main components. Our research consists of two main parts. The first part includes general demographic information, a total of 17 items, including gender, age, place of residence, family income, anemia, heart failure (HF), serum calcium marital status, hypertension [Meet the diagnostic criteria for hypertension established by the Chinese Guidelines for the Prevention and Treatment of Hypertension (2024 Revision) (28)], diabetes [meet the diagnostic criteria for diabetes (29)], etc.

#### Generalized anxiety disorder scale

The 7-item Generalized Anxiety Disorder scale (GAD-7) (30), developed by Spitzer et al., was employed to assess participants’ anxiety levels during the preceding two-week period. This validated instrument comprises seven items, each scored on a 4-point Likert scale ranging from 0 (“not at all”) to 3 (“nearly every day”), yielding a total score range of 0-21. Higher total scores indicate greater anxiety severity, with established clinical cutoffs as follows: 0-4 (minimal/normal), 5-9 (mild), 10-14 (moderate), and 15-21 (severe). In this study, the Cronbach’s α for the sample was 0.852.

#### Lubben social network scale

The Lubben Social Network Scale was originally compiled by Lubben in 1988 (31) and has since been revised with six entries (LSNS-6) (32). Consists of the family network (1–3 questions) and friend network (4–6 questions) two parts, the total score of 0–30 points, the smaller the score, the higher the degree of social isolation, < 12 points indicates the existence of social isolation (33). In this study, the Cronbach’s α for the sample was 0.921.

### Data collection

Before commencing the survey, all investigators underwent standardized training via video sessions that covered survey principles, procedural precautions, and guidance on providing clear explanations. During the survey, investigators engaged patients in face-to-face interactions, presenting paper-based questionnaires to elucidate the survey’s purpose, content, and completion process. Upon obtaining patient consent, questionnaires were distributed, allowing patients to complete them independently independently. Completed questionnaires were promptly retrieved on-site. In instances where patients were unable to complete the questionnaire due to unique circumstances, investigators impartially presented the questions and assisted in truthful completion.

### Sample size

Sample size was calculated based on the principle that event per variable (EPV) ≥ 10 (34). 17 variables were expected to be included in this study, considering a loss to follow-up rate of 10%-20%, so the minimum sample size for modeling was 210. Since the modeled sample size represents 70% of the total sample, the total sample size is at least 300. A total of 370 questionnaires were distributed, with 362 effectively collected, yielding a robust retrieval rate of 97.837%.

### Statistical analysis

Statistical analyses were conducted using SPSS 27.0, with categorical variables presented as frequencies and percentages (n, %). Between-group comparisons were performed via Chi-square tests, and potential risk factors were identified through logistic regression analysis (P < 0.05). The dataset was split into a training set (70%) and a testing set (30%). To address class imbalance in the training data, we applied the Synthetic Minority Over-sampling Technique (SMOTE) to generate synthetic instances of the minority class and utilized algorithmic class weighting by setting class_weight=‘balanced’ where applicable. The preselected features were subsequently used to train seven machine learning models: LR, EN, KNN, DT, XGB, SVM, and RF. For each model, hyperparameter tuning was performed using 5-fold cross-validation and grid search to maximize training set accuracy, thereby ensuring optimal performance and reliable prediction on the test set. All models underwent five-fold cross-validation to enhance robustness and reliability. Model comparison was based on AUC values, from which the best-performing model was selected. Finally, we employed SHapley Additive exPlanations (SHAP) analysis. The SHAP algorithm—optimized for tree ensemble models—was used to calculate the SHAP value of each predictor for every sample in the test set, reflecting its contribution to individual predictions. Subsequently, a SHAP summary plot was generated to visually illustrate both the overall importance ranking of features (represented by point density) and the dependency trends between SHAP values and feature magnitudes.

## Results

### General characteristics of MHD participants

A total of 362 MHD participants were included, comprising 238 males (65.7%) and 124 females (34.3%). Among these participants, 166 (45.856%) were diagnosed as socially isolated, while the remaining 196 (54.143%) did not show any signs of social isolation. Detailed demographic and clinical characteristics are presented in **Table 1**.

**Table 1:** General characteristics of MHD participants.

Variables	Total	No	Yes	χ²	*P*
Gender				1.304	0.253
Male	238	134	104		
Female	124	62	62		
Age(Years)				62.228	<0.001
18-44	60	9	51		
45-59	105	48	57		
60-74	158	114	44		
>74	39	25	14		
Educational background				6.463	0.091
Primary school or below	26	18	8		
Junior high school	172	97	75		
Senior high school	144	68	76		
College or above	20	13	7		
Place of Residence				148.584	<0.001
Rural	241	11	110		
Urban	121	185	56		
Monthly household income(￥)				69.513	<0.001
≤2000	96	18	78		
2001-4000	113	68	45		
≥4001	153	110	43		
Dialysis vintage(Years)				11.943	0.008
1-3	73	32	41		
3-5	72	38	34		
5-7	54	40	14		
>7	163	86	77		
Sleep condition				30.501	<0.001
Good	218	140	78		
Fair	110	50	60		
Poor	34	6	28		
Medication categories				5.163	0.023
1–2 types	99	44	55		
3–4 types	263	152	111		
History of diabetes				1.462	0.227
No	77	37	40		
Yes	285	159	126		
Hypertension history				0.878	0.349
No	34	21	13		
Yes	328	175	153		
Anemia				8.616	0.003
No	128	56	72		
Yes	234	140	94		
Heart failure (HF)				86.928	<0.001
No	215	73	142		
Yes	147	123	24		
Serum calcium				1.579	0.454
2.25~2.75mmol/L	172	92	80		
<2.25 mmol/L	179	96	83		
>2.75 mmol/L	11	8	3		
C-reactive protein (CRP)				5.041	0.025
≤8 mg/L	227	159	118		
>8 mg/L	85	37	48		
Total cholesterol				1.365	0.243
<6.2mmol/L	357	192	165		
≥6.2mmol/L	5	4	1		
Character				0.600	0.439
Introverted	173	90	83		
Outgoing	189	106	83		
Anxiety				26.532	<0.001
No	244	155	89		
Yes	118	41	77		

### Logistic regression analysis

The incidence of social isolation in MHD patients was taken as the dependent variable (No=0, Yes=1). Based on age (> 74 years old =0, 60–74 years old =1, 45–59 years old, 18–43 years old =3), Sleep condition (good =0, fair =1, poor =2), HF (no =0, yes =1), Monthly household income (≥4000￥=0, 2000-3999￥=1, < 2000￥=2), anxiety (no =0, Yes =1), Place of Residence (Rural=0, Urban=1), Dialysis vintage (> 7Years=0) 5–7 Years=1, 3–5 years =2, 1–3 years =3), Medication categories (1–2 types=0, 3–4 types=1), Anemia (No=0, Yes=1) CRP (≤8 mg/L = 0, > 8 mg/L = 1) was taken as the independent variable and logistic regression analysis was conducted. A total of 6 risk factors were identified through analysis (P < 0.05), as shown in **Table 2**.

**Table 2:** Logistic regression analysis.

Risk factor	Reference factor	B	SE	Waldx^2^	P	OR	95%CI
Age	>74 Years						
18–44 Years		3.077	0.848	13.172	<0.001	21.687	4.117-114.237
Place of Residence	Rural						
Urban		3.005	0.535	31.481	<0.001	20.177	7.064-57.632
Sleep condition	Good						
Poor		1.873	0.727	6.637	0.010	6.507	1.565-27.052
HF	No						
Yes		2.542	0.448	32.209	<0.001	12.707	5.282-30.572
Anxiety	No						
Yes		2.096	0.462	20.573	<0.001	8.136	3.289-20.129
Monthly household income(￥)	≥4000						
<2000		2.542	0.448	32.209	<0.001	12.707	5.282-30.572

### Performance evaluation

**Figure 1:**
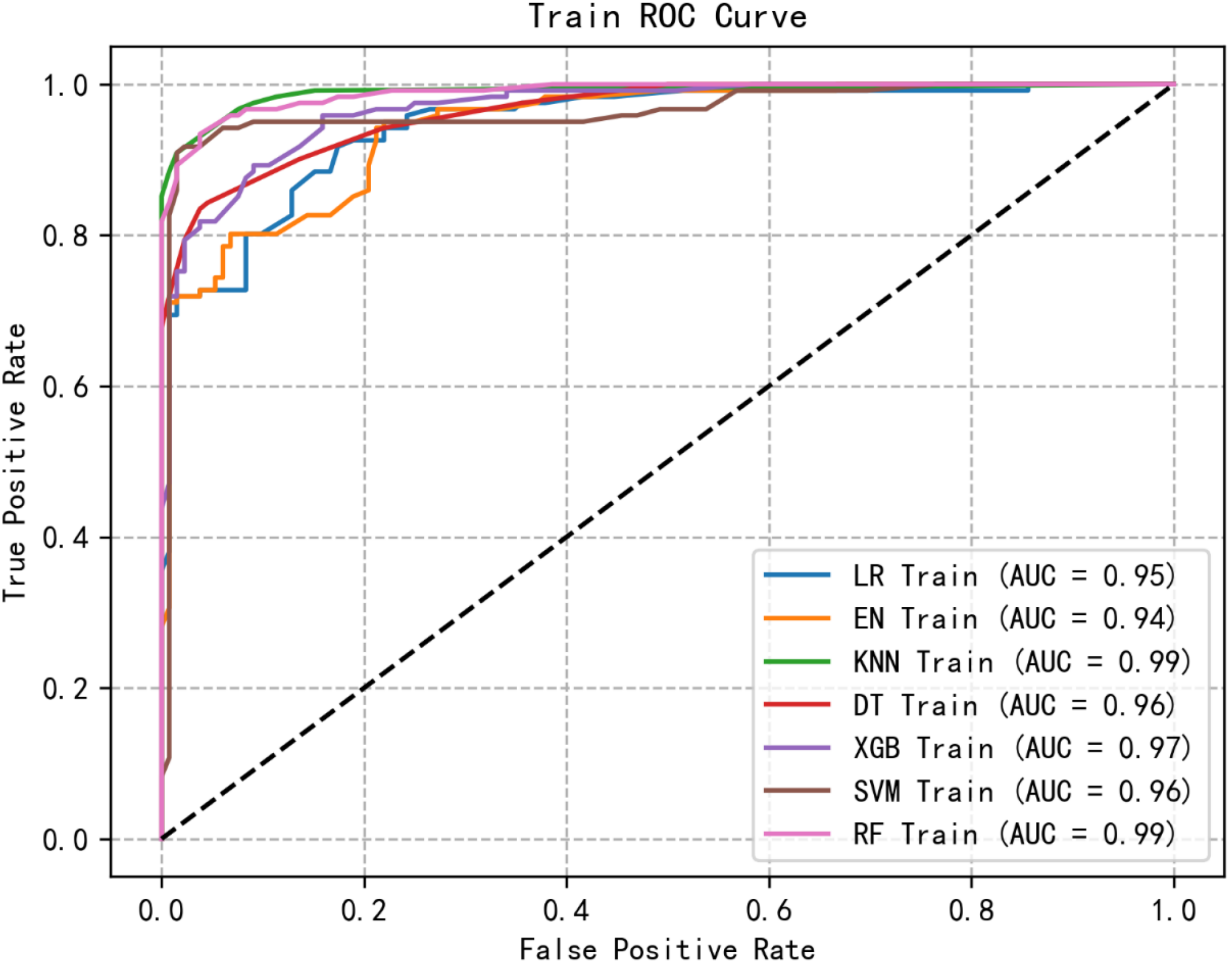
ROC curve of the training group of machine learning algorithms.

**Figure 2:**
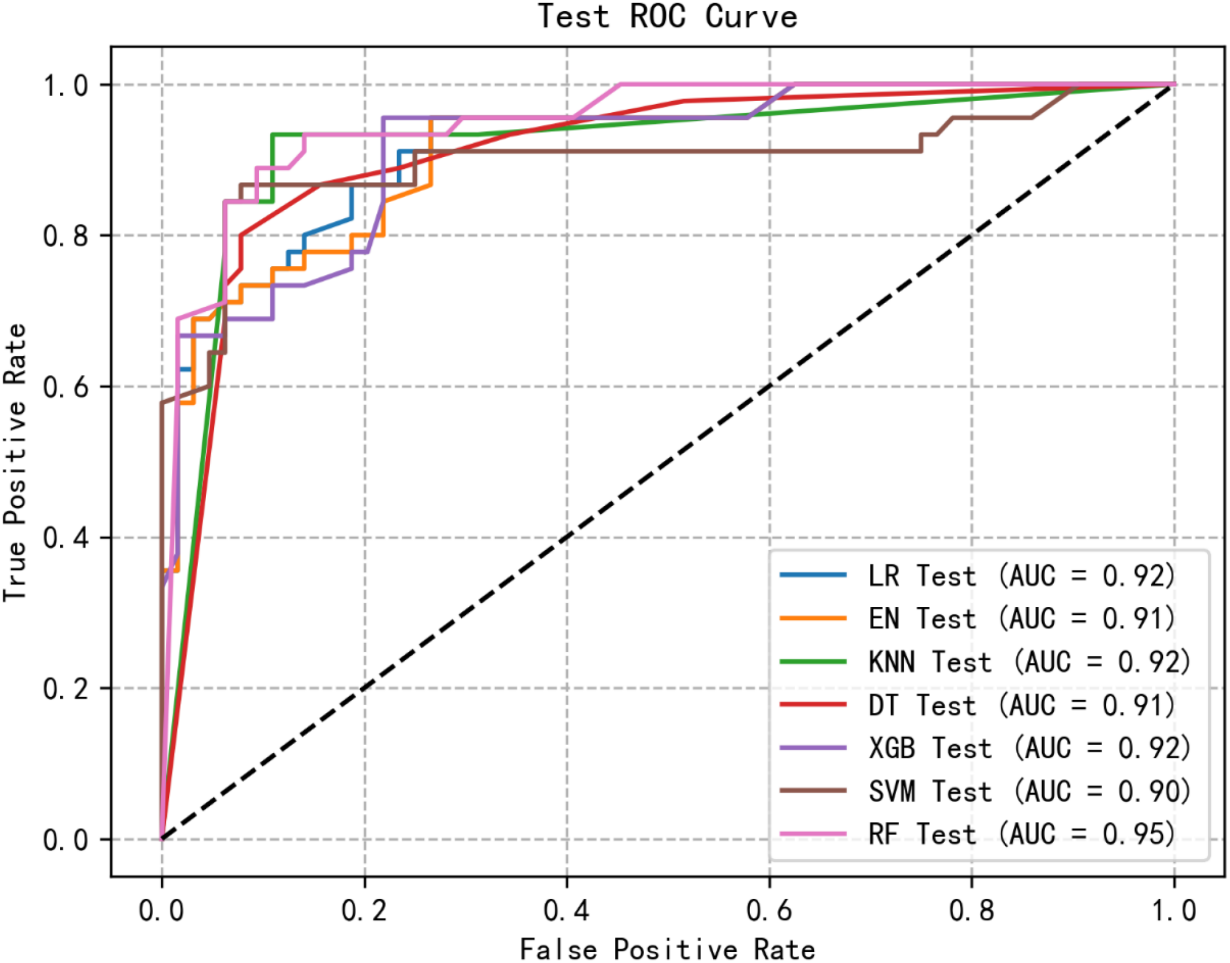
ROC curve of test group of machine learning algorithms.

**Figure 3:**
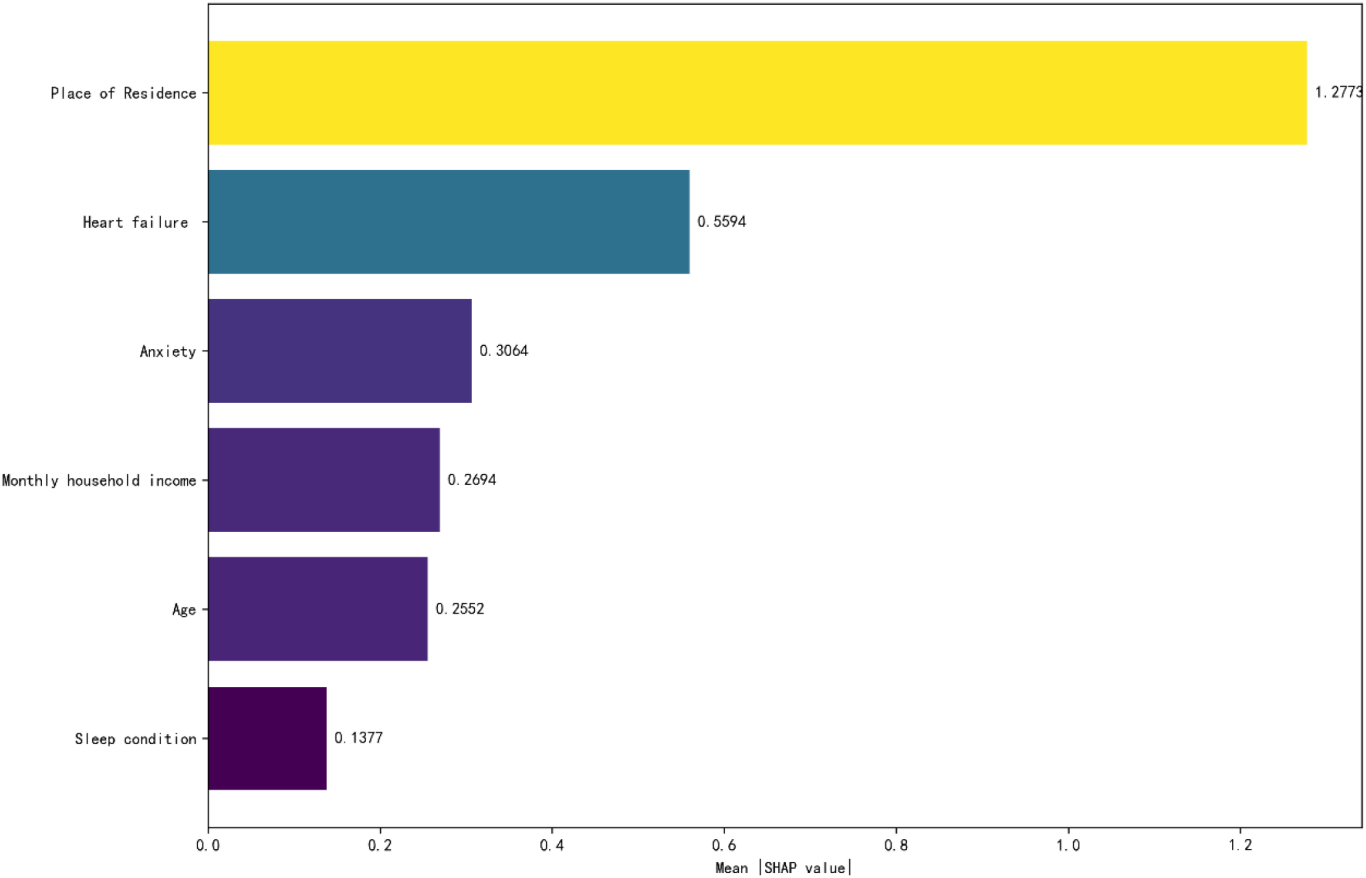
Feature importance ranking.

**Figure 4:**
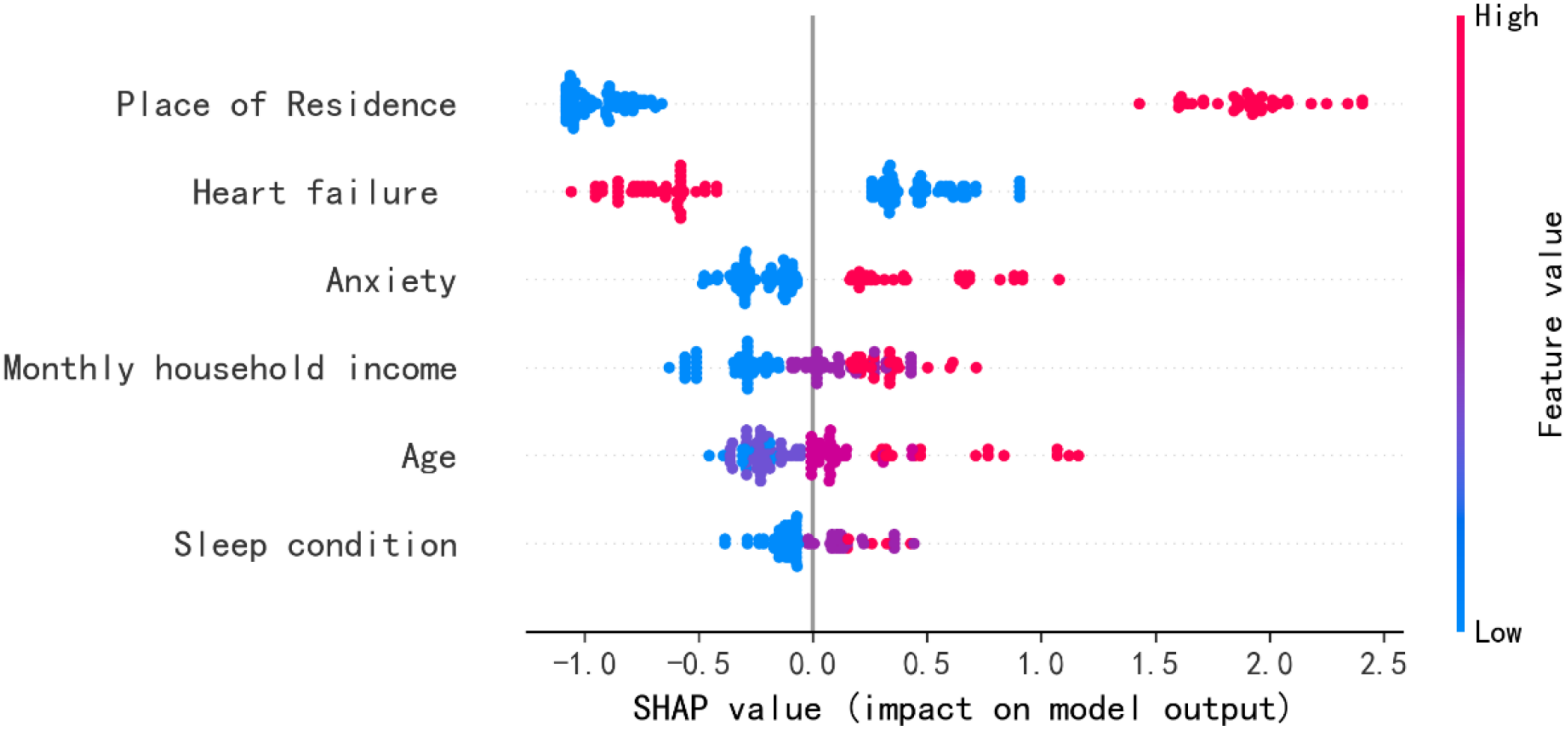
SHAP value (impact on model output).

Through logistic regression analysis, six significant and highly correlated individual characteristics were identified and retained. The dataset was partitioned into training and test sets, with cross-validation performed exclusively on the training set. Subsequently, seven ML models were developed to predict the risk of social isolation in patients undergoing MHD. **Figures 1**, **2** show the receiver operating characteristic (ROC) curves of all models in the test set and validation set, and their predictive performance is quantified by the area under the curve (AUC). Among the results of the test set, the AUC of the RF model was the highest (0.72), followed by XGB (0.92), KNN (0.92), LR (0.92), EN (0.91), DT (0.91), SVM (0.90). Furthermore, we evaluated the accuracy, sensitivity, specificity and confusion matrix of each model (**Table 3**, **Appendix 1**: **Figures 1-7**). The RF model demonstrated the highest accuracy (0.881), and when combined with other performance indicators, it showed the most robust overall performance.

**Table 3:** Comparison of prediction models.

Prediction model	Accuracy	Precision	Recall	F1	Sensitivity	Specificity	Kappa
LR training group	0.862	0.898	0.802	0.847	0.801	0.917	0.722
LR test group	0.826	0.795	0.778	0.786	0.778	0.859	0.639
EN training group	0.862	0.898	0.802	0.847	0.802	0.917	0.722
EN test group	0.826	0.795	0.778	0.787	0.778	0.859	0.639
KNN training group	0.949	0.982	0.909	0.944	0.909	0.985	0.897
KNN text group	0.872	0.844	0.844	0.844	0.844	0.891	0.735
DT training group	0.901	0.944	0.843	0.891	0.843	0.955	0.801
DT text group	0.871	0.878	0.800	0.837	0.800	0.922	0.732
XGB training group	0.897	0.893	0.893	0.893	0.893	0.902	0.794
XGB text group	0.826	0.740	0.889	0.808	0.889	0.781	0.651
SVM training group	0.949	0.982	0.909	0.944	0.909	0.985	0.897
SVM text group	0.862	0.813	0.867	0.839	0.867	0.859	0.719
RF training group	0.949	0.958	0.933	0.946	0.934	0.962	0.897
RF text group	0.881	0.820	0.911	0.863	0.911	0.859	0.758

### Feature importance ranking

The factors influencing social isolation in MHD patients are: Place of residence (1.277), HF (0.559), Anxiety (0.306), Monthly household income (0.269), Age (0.255), Sleep condition (0.138). A negative SHAP value (in absolute terms) indicates that the feature has a negative impact on social isolation, meaning that a higher value of this feature leads the prediction toward the negative class. Conversely, a positive SHAP value (in absolute terms) suggests that the feature has a positive impact on social isolation, implying that a higher value of this feature directs the prediction toward the positive class. Conversely, a positive SHAP value (in absolute terms) suggests that the feature has a positive impact on social isolation, implying that a higher value of this feature directs the prediction toward the positive class, see **Figures 3**, **4**.

## Discussion

This study applied seven ML methods to investigate the factors associated with social isolation in patients on MHD, in which the RF model demonstrated superior predictive performance. Compared to conventional statistical models (6), Random Forest effectively captures nonlinear relationships among variables while maintaining strong stability in both classification and regression tasks (35). Moreover, the inherent stochasticity of the model enhances generalization capacity and mitigates the risk of overfitting (36). This RF-based approach enables timely identification of high-risk individuals, facilitating targeted interventions to mitigate adverse outcomes and slow social isolation progression in this population.

Our research results show that the incidence of social isolation in MHD patients is 46.856%, which is significantly higher than that in stroke patients (27.7%) and diabetic patients (31.2%) (37, 38). MHD patients generally exhibit poor overall health. Prominent post-dialysis fatigue and sleep disturbances, combined with reduced skeletal muscle mass and function, significantly impair physical activity and mobility (39). Frequent medical visits further limit their social engagement. Additionally, psychological comorbidities such as anxiety and depression decrease outings and social interactions, progressively narrowing their social circles and elevating the risk of social isolation (6). Social isolation substantially diminishes interpersonal communication, perpetuating a state of prolonged detachment. This condition may trigger detrimental physical and psychological consequences (4). Therefore, identifying risk factors for social isolation in MHD patients is clinically imperative. In this study, we first employ logistic regression to screen potential risk factors and subsequently apply a random forest model to quantify their relative importance. The following risk factor levels were analyzed and obtained: Place of residence (1.277), HF (0.559), Anxiety (0.306), Monthly household income (0.269), Age (0.255), Sleep condition (0.138). These findings provide actionable goals for clinicians to conduct active social isolation screening and intervention in the MHD population.

Our study identifies residence location as a primary determinant of social isolation among MHD patients, with rural patients exhibiting significantly higher rates of isolation than their urban counterparts. This disparity likely stems from urban areas’ advantages in transportation networks, economic resources, and infrastructure development. Rural patients face compounded challenges: limited health information access, culturally constrained disease awareness, and reduced economic capacity to obtain equitable medical services (40). These constraints not only impose physical and financial burdens—including high dialysis costs—but also exacerbate perceived stigma. Such stigma fosters adverse psychosocial outcomes, including shame, embarrassment, diminished self-worth, and withdrawal behaviors (41). To cope, patients often conceal their condition and restrict social interactions, further impairing relationship-building and societal reintegration (42). To mitigate these effects, dual interventions are critical. First, clinicians should support patients in accepting their disease to prevent social withdrawal. Second, public education campaigns targeting patients’ social networks and communities can improve disease understanding, reduce discrimination, and enhance patients’ confidence in re-engaging with society.

Anxiety significantly compromises the mental health of MHD patients, leading to social withdrawal and exacerbating their social alienation. Current evidence indicates that dialysis-related physical complications can impair patients’ psychological well-being, diminishing their interest in social activities and disrupting everyday social interactions (43). Nagy et al (44). further demonstrated that anxiety and other negative emotional states increase psychological vulnerability, reducing patients’ willingness to engage socially and intensifying their social isolation. Notably, MHD patients face a 20-fold increased risk of cardiovascular diseases compared with the general population, with HF representing the second most prevalent cardiovascular complication (prevalence ≈1.1%) (45). Those with concurrent HF experience a symptom burden comparable to cancer patients (46), where greater symptom load is associated with functional decline, including reduced independence and impaired activities of daily living (47). The consequent need for frequent hospitalizations further restricts patients’ mobility (48), which in turn limits opportunities for social engagement and corresponds with a higher likelihood of social isolation.

Our study identified monthly household income as a significant determinant of social isolation in MHD patients, a finding consistent with the work of Umarova et al (49). Patients with higher incomes exhibited greater diversity in social relationships, more disposable resources, and more active life participation. In comparison, patients with lower incomes presented multiple burdens—economic, psychological, and physical—during dialysis treatment, factors associated with increased social withdrawal. While approximately 60% of patients received subsidies, dialysis-related expenses, including both direct medical costs and indirect burdens such as transportation and time commitments, remained a substantial challenge, correlated with heightened negative emotions and feelings of social alienation (50). Furthermore, our results confirm previous observations that middle-aged patients face a significantly higher risk of social isolation than elderly patients (51). As primary family providers with strong self-worth expectations, these patients are particularly vulnerable to the social limitations imposed by long-term hemodialysis. Compounding this vulnerability, 46-85% of MHD patients experience sleep disorders (52, 53), a condition associated with physical and mental dysfunction, reduced quality of life, and elevated mortality. Dialysis-related symptoms and prolonged nighttime wakefulness exacerbate fatigue, accelerate cognitive decline, and further diminish social participation motivation (54). These findings underscore the need for targeted interventions, as clinicians should prioritize middle-aged patients for self-management training and peer support programs to enhance their confidence. Simultaneously, income-limited patients and those with sleep disturbances require focused screening. Policy improvements should expand social security provisions while optimizing dialysis center environments to promote restful sleep, thereby mitigating social alienation.

## Strengths and limitations

This study aimed to develop and validate a machine learning model for predicting the risk of social isolation in MHD patients. The study systematically collected multidimensional clinical and sociodemographic variables and incorporated a validated social isolation assessment scale, focusing on the MHD population with clear clinical relevance and management implications. The Random Forest model demonstrated favorable predictive performance in testing, offering evidence-based insights to support early identification and targeted intervention for social isolation in this patient population. Several limitations should be noted. First, the absence of genomic data constrains mechanistic interpretation. As a single-center cross-sectional study with a moderate sample size, the generalizability of findings may be limited. Future multicenter longitudinal studies with larger cohorts are needed to validate these results and identify objective biomarkers for social isolation in this population. In this study, a repeated random sampling approach was applied during cross-validation. However, constrained by our current sample size, implementing k-fold nested cross-validation would result in a substantial reduction in the size of the inner-loop training set. This could potentially increase the risk of underfitting and lead to unstable performance evaluation with higher variance. Future studies involving external validation using larger independent cohorts will be essential to definitively assess the model’s generalizability. As datasets expand, it is recommended to adopt more robust validation methods, such as nested cross-validation, to enhance the reliability and reproducibility of model evaluation. This study’s coding approach for predictor variables may not adequately capture the true underlying data structure. Future research, if equipped with higher-precision data, could employ continuous variables or nonlinear basis functions in modeling to more finely investigate the association patterns between variables and the outcome. The sample size in this study is adequate for the initial development and comparison of multiple predictive models; however, the model performance, especially for more complex models, requires external validation in larger, independent prospective cohorts to confirm its generalizability. As a convenience sampling method was utilized in our study, selection bias may have been introduced, potentially limiting the generalizability of the proposed model. Future studies are therefore recommended to incorporate multi-center, large-sample investigations to enhance sample representativeness and improve the model’s generalizability and external validity. Finally, the data for our study were collected from a single medical center, and the participants shared relatively homogeneous cultural backgrounds, social support systems, and health perceptions. As a result, the representativeness of the sample is limited, and caution should be exercised when generalizing the findings to other centers or populations with different hospital management policies, resource allocation, clinical protocols, or sociocultural contexts.

## Conclusion

In this study, seven machine learning algorithms were employed to develop a risk prediction model for social isolation in MHD patients, with the random forest model exhibiting the best predictive effect. The key risk factors were analyzed and identified: place of residence, heart failure (HF), Anxiety, Monthly household income, Age, and Sleep condition. These findings enable clinicians to implement targeted screening and personalized intervention for high-risk MHD patients. Although it provided clinically actionable insights, this study has inherent limitations, including a single-center, cross-sectional design and limited sample size. Future multicenter longitudinal studies require larger cohorts to enhance universality. Machine learning, as the core technology of artificial intelligence, possesses robust data processing capabilities and holds special application prospects in disease risk prediction. The continuous development of advanced algorithms will further enhance the social frailty prediction model for the MHD population, ultimately promoting the early identification and intervention of this vulnerable patient group.

## Data Availability

The original contributions presented in the study are included in the article/**Supplementary Material**. Further inquiries can be directed to the corresponding author.
