# Supplementary figures and images for: Predicting social isolation in maintenance hemodialysis patients using machine learning methods: a cross-sectional study
Source: Front Psychiatry. 2026 Feb 18;17:1776298. doi: 10.3389/fpsyt.2026.1776298 (PMC12957182; doi:10.3389/fpsyt.2026.1776298)

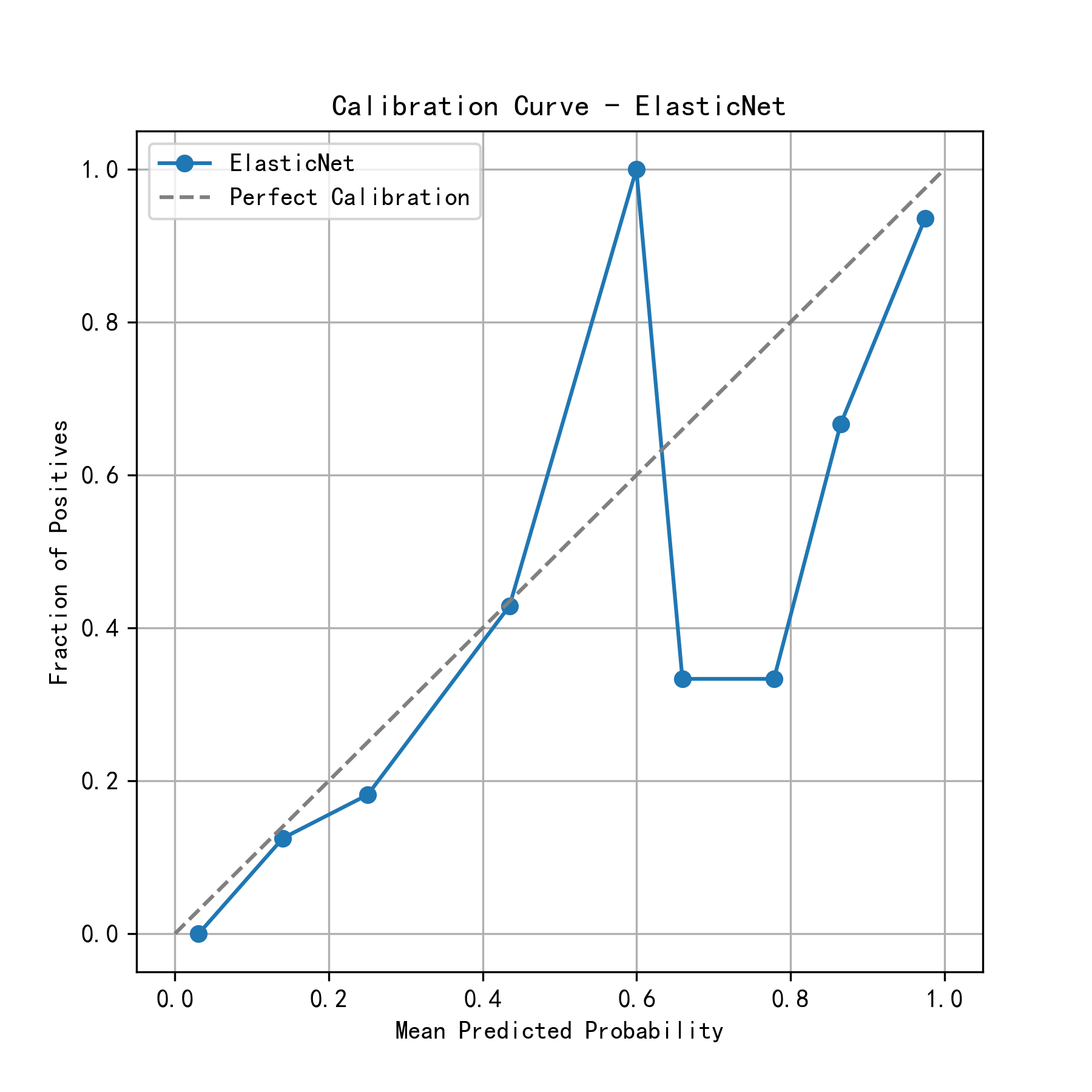

Supplement: Supplementary file 1 [file DataSheet1.zip › Supplementary material/Fifure 11.Calibration Curve-ElasticNet .png]

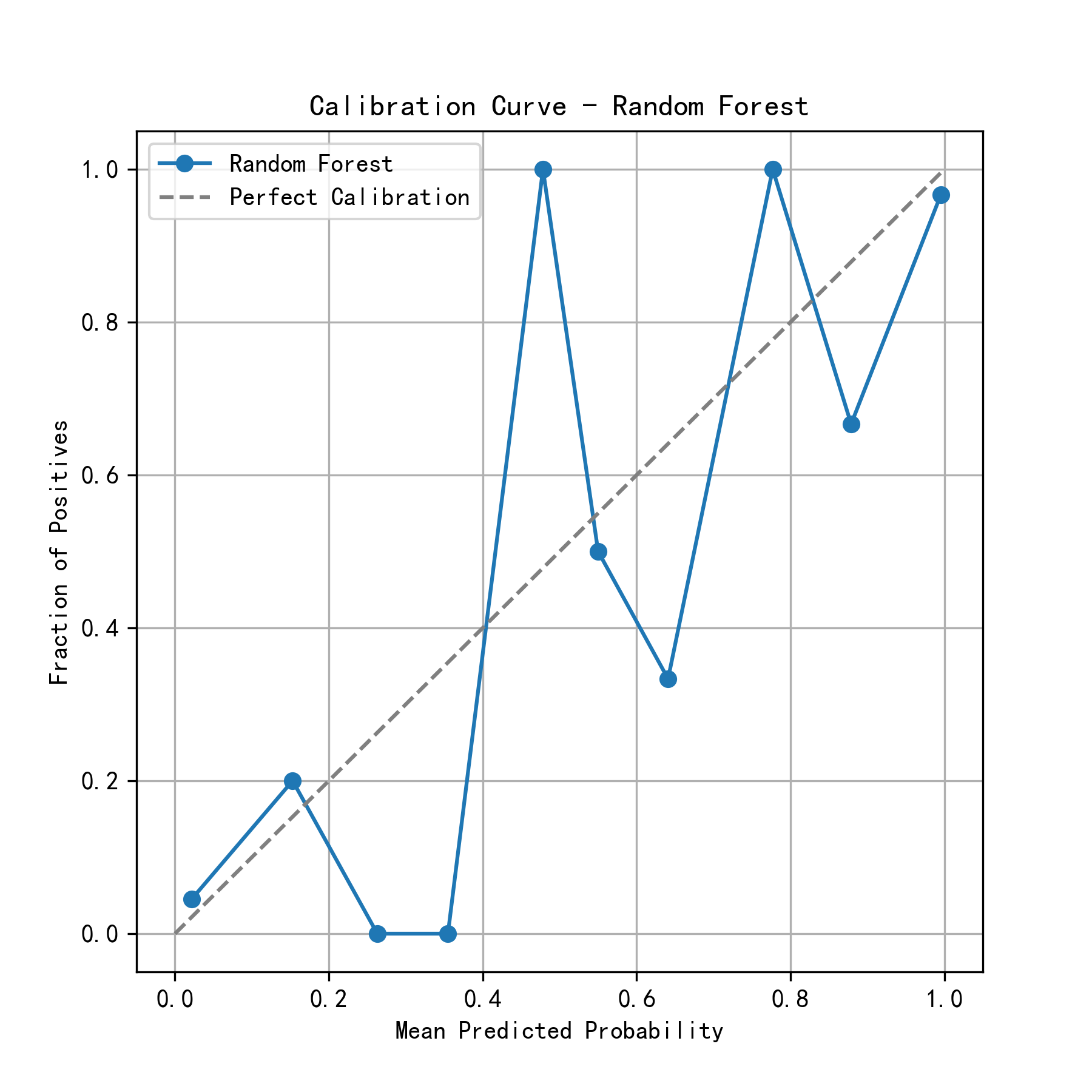

Supplement: Supplementary file 1 [file DataSheet1.zip › Supplementary material/Fifure 14.Calibration Curve- Random Forest.png]

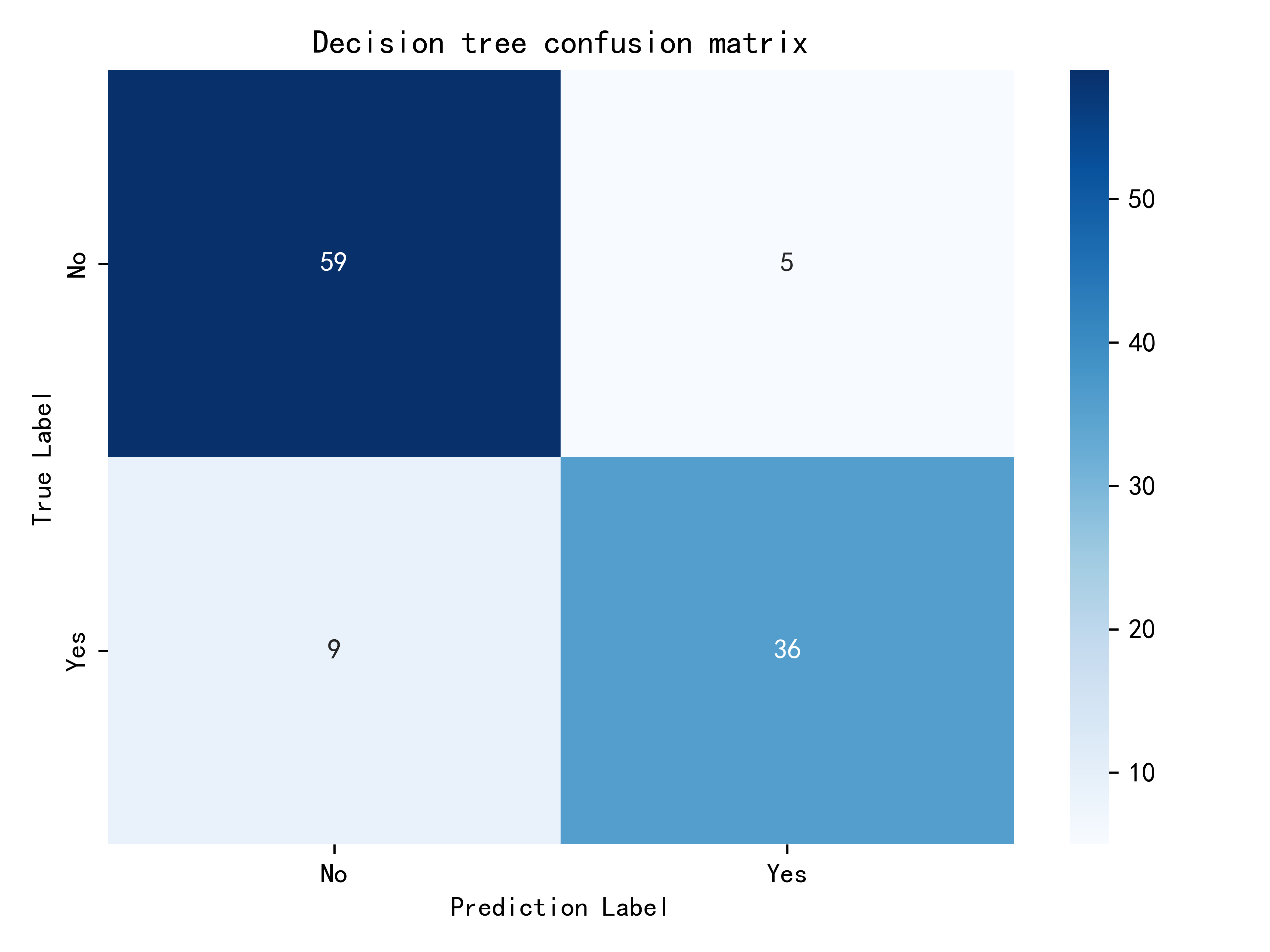

Supplement: Supplementary file 1 [file DataSheet1.zip › Supplementary material/Figure 1.Decision tree.png]

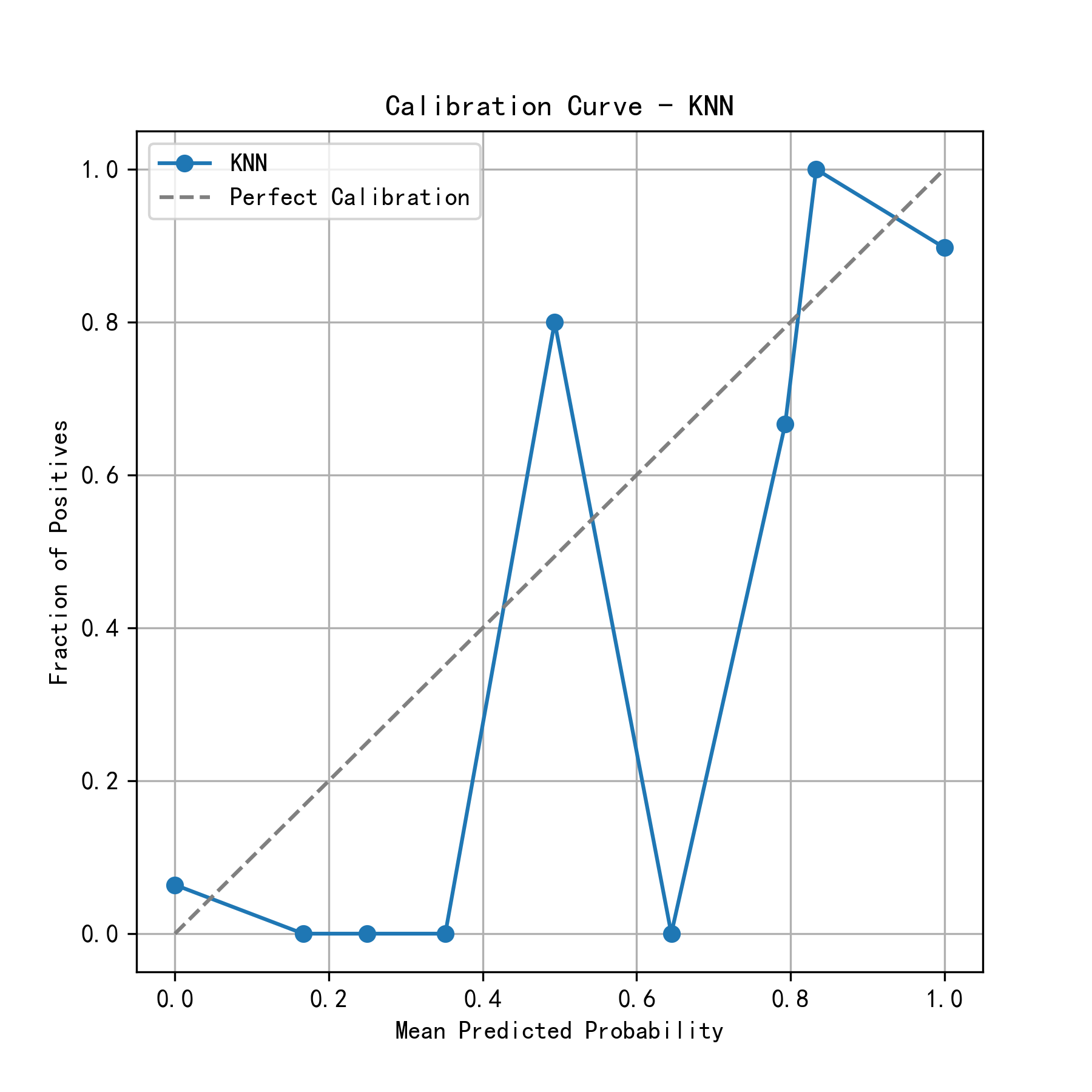

Supplement: Supplementary file 1 [file DataSheet1.zip › Supplementary material/Figure 10Calibration Curve -KNM.png]

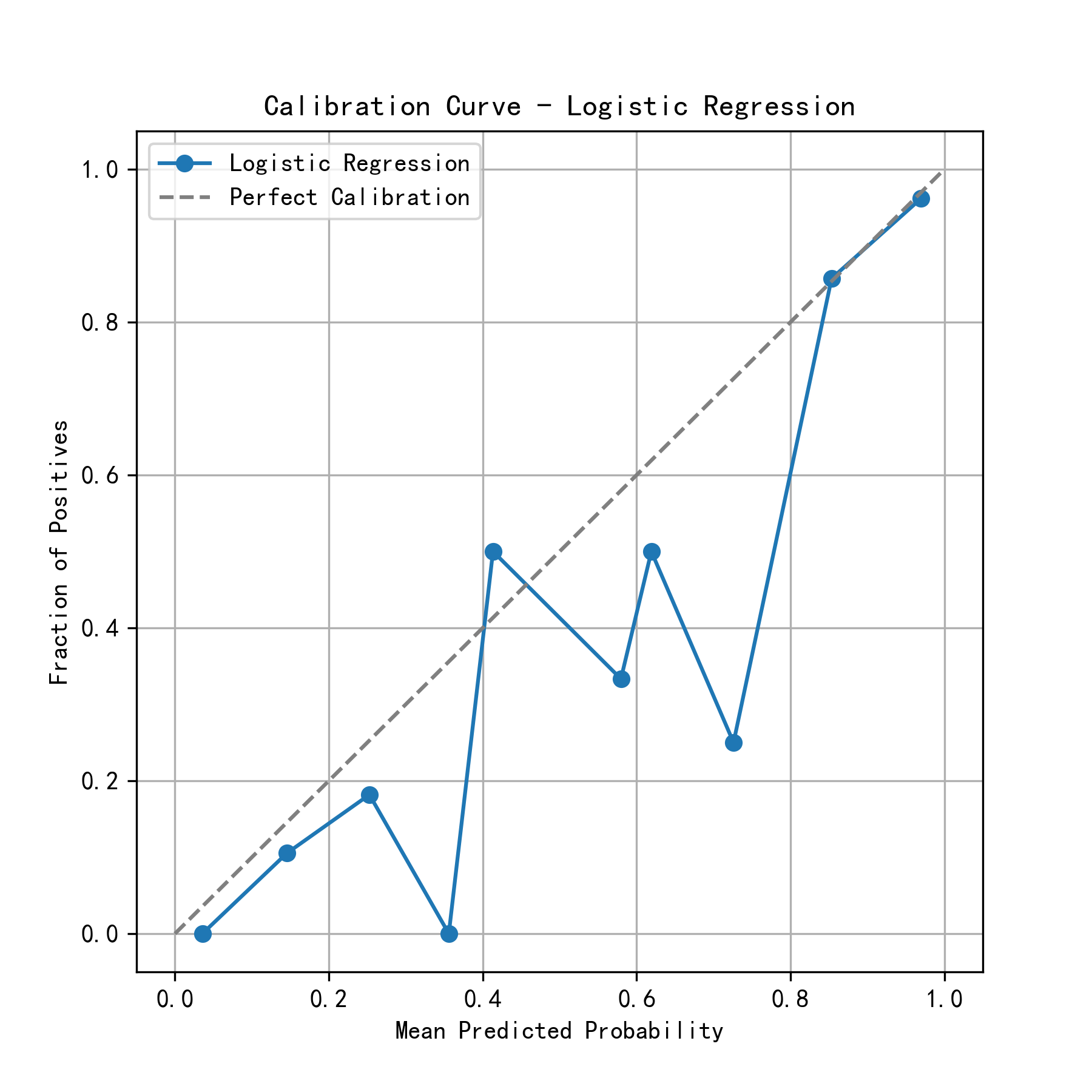

Supplement: Supplementary file 1 [file DataSheet1.zip › Supplementary material/Figure 12.Calibration Curve-Logistic Regression.png]

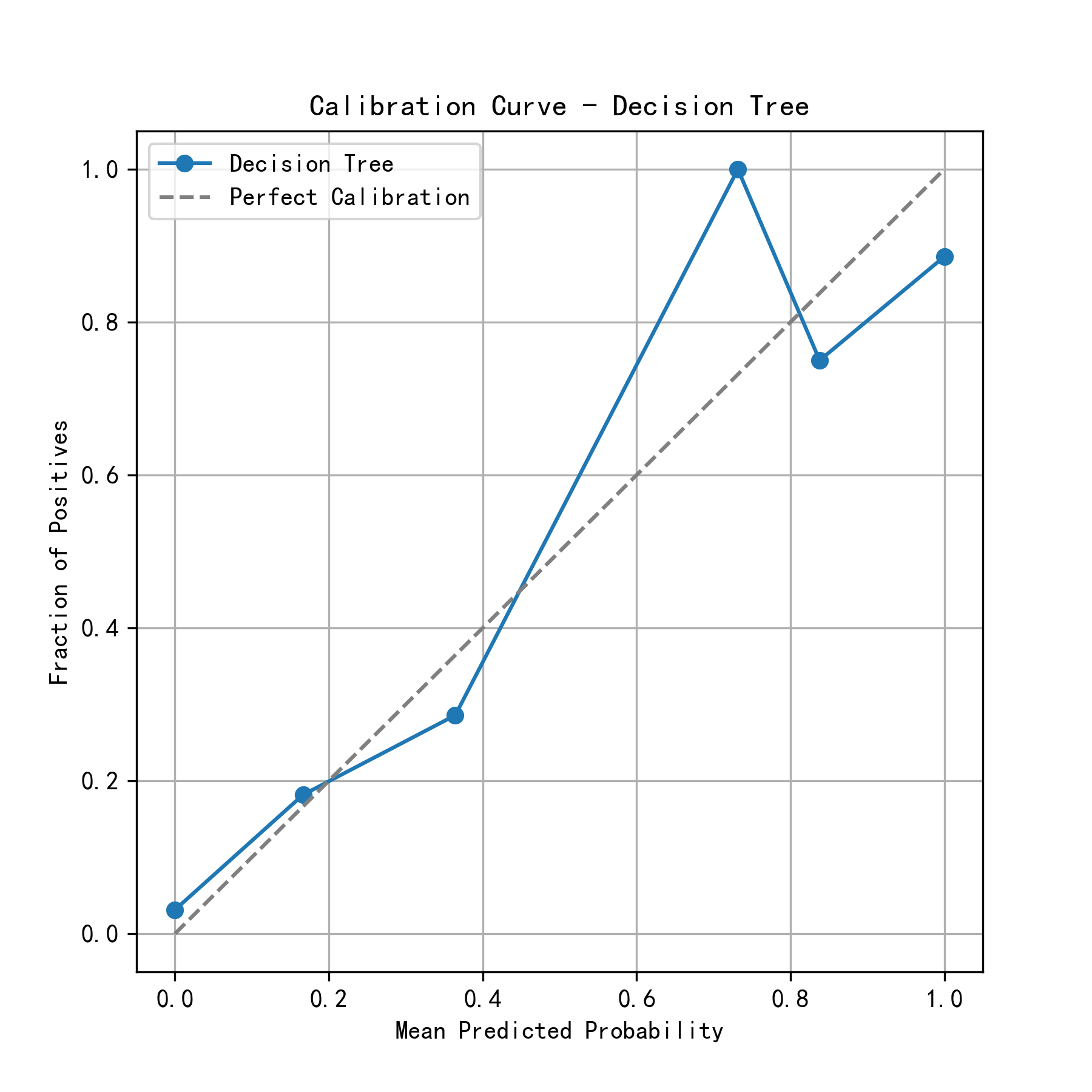

Supplement: Supplementary file 1 [file DataSheet1.zip › Supplementary material/Figure 13.Calibration Curve-Decision Tree.png]

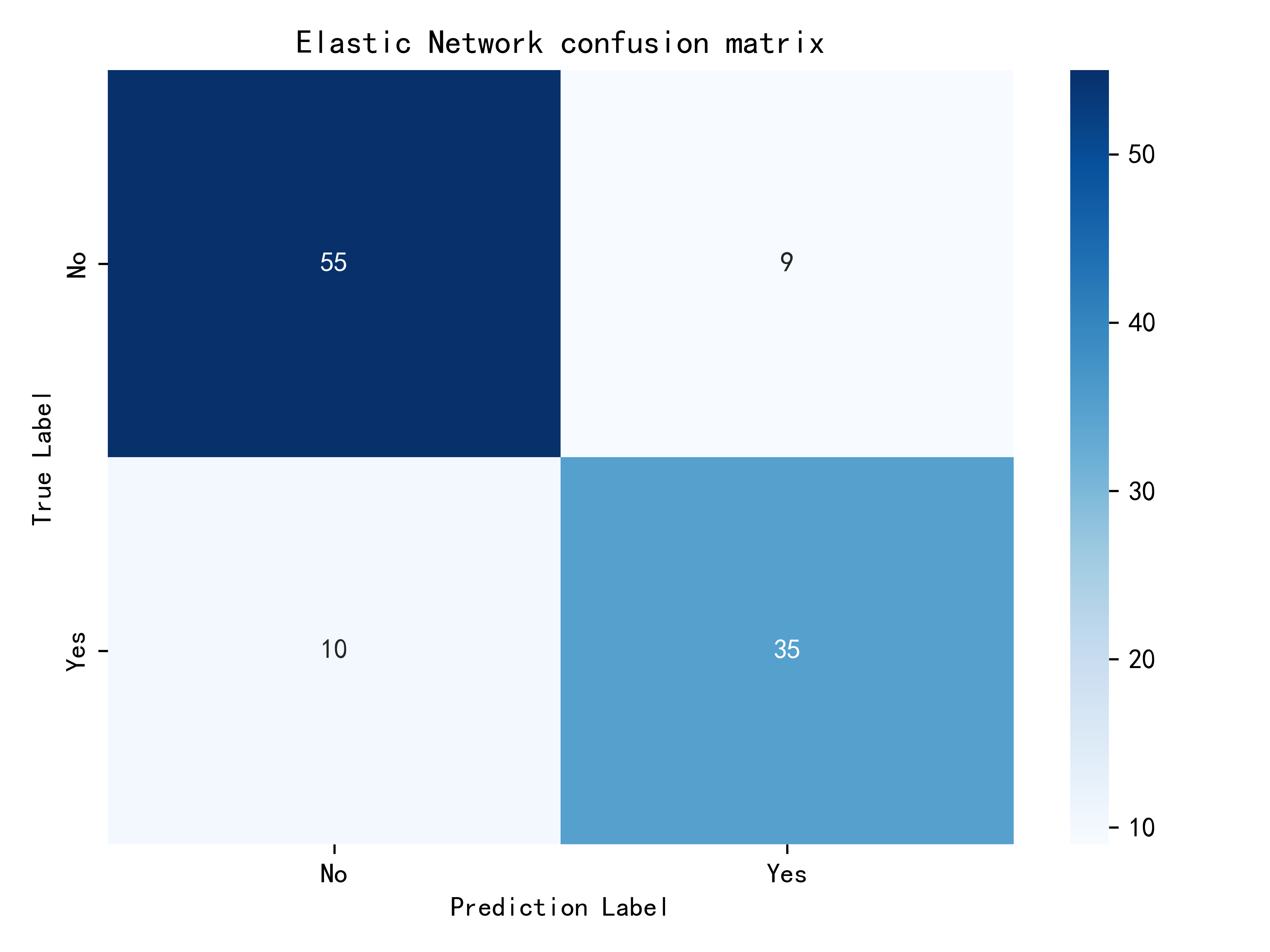

Supplement: Supplementary file 1 [file DataSheet1.zip › Supplementary material/Figure 2.Elastic Network.png]

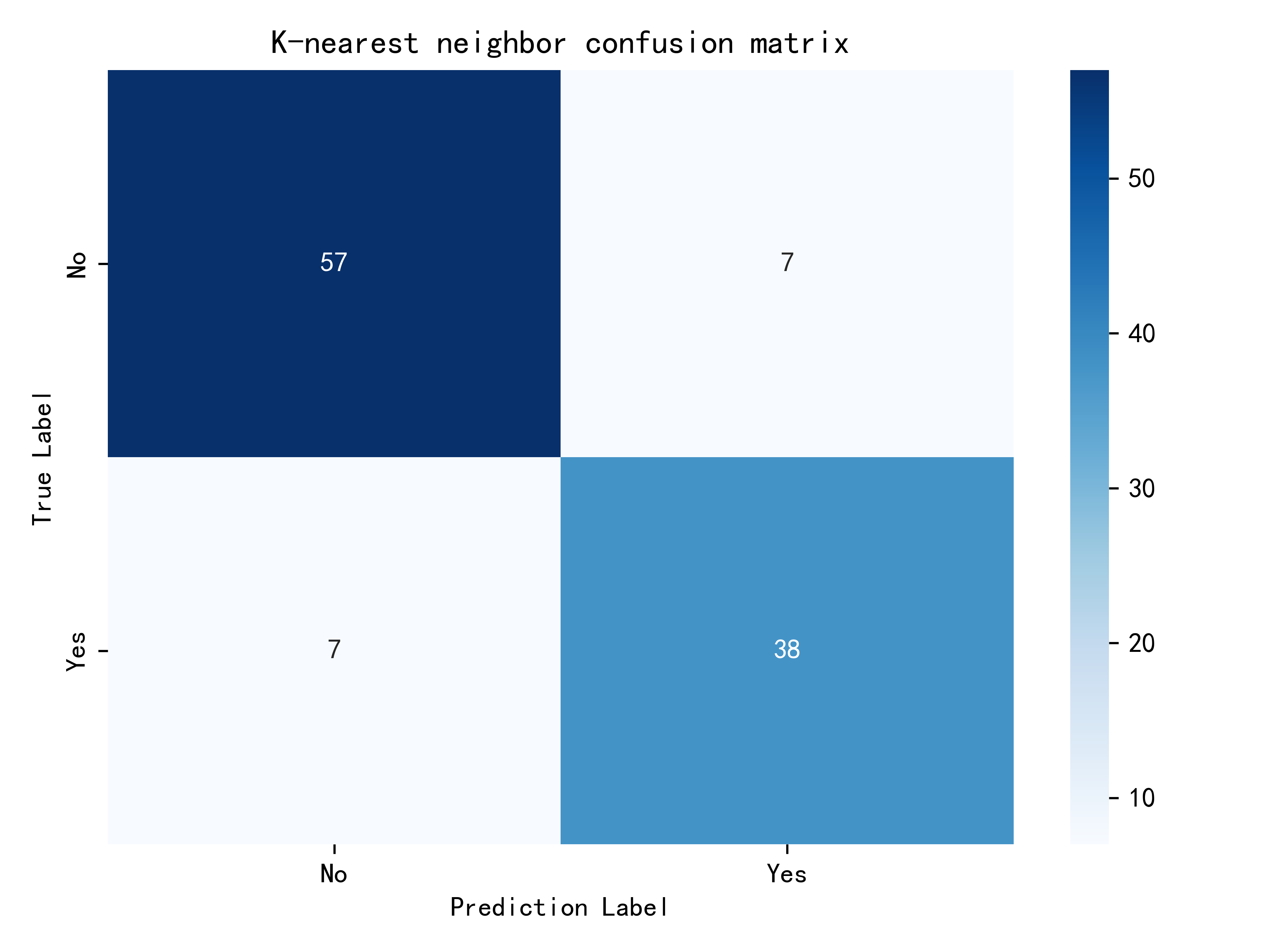

Supplement: Supplementary file 1 [file DataSheet1.zip › Supplementary material/Figure 3.K-nearest neighbor.png]

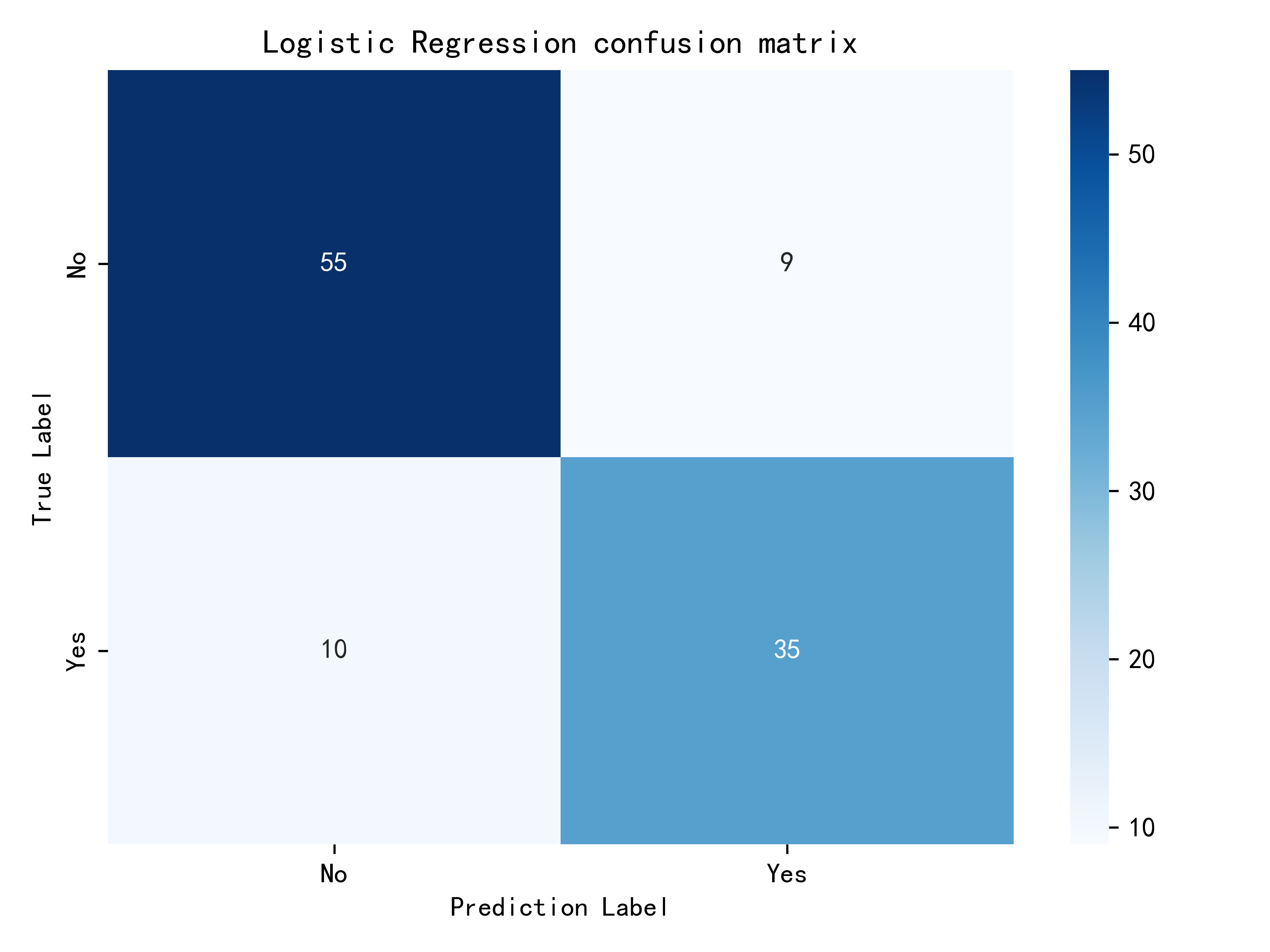

Supplement: Supplementary file 1 [file DataSheet1.zip › Supplementary material/Figure 4.Logistic Regression.png]

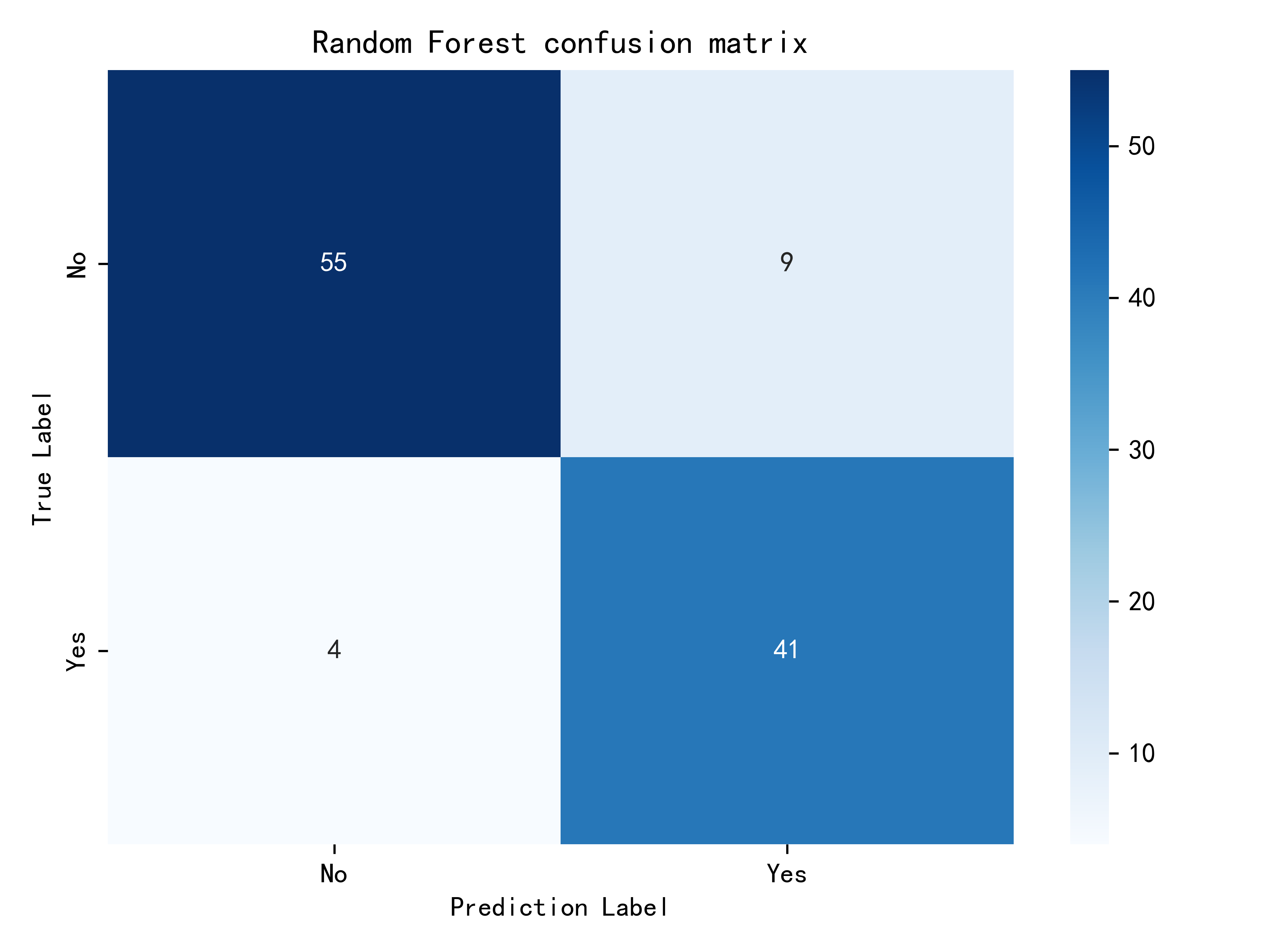

Supplement: Supplementary file 1 [file DataSheet1.zip › Supplementary material/Figure 5.Random Forest.png]

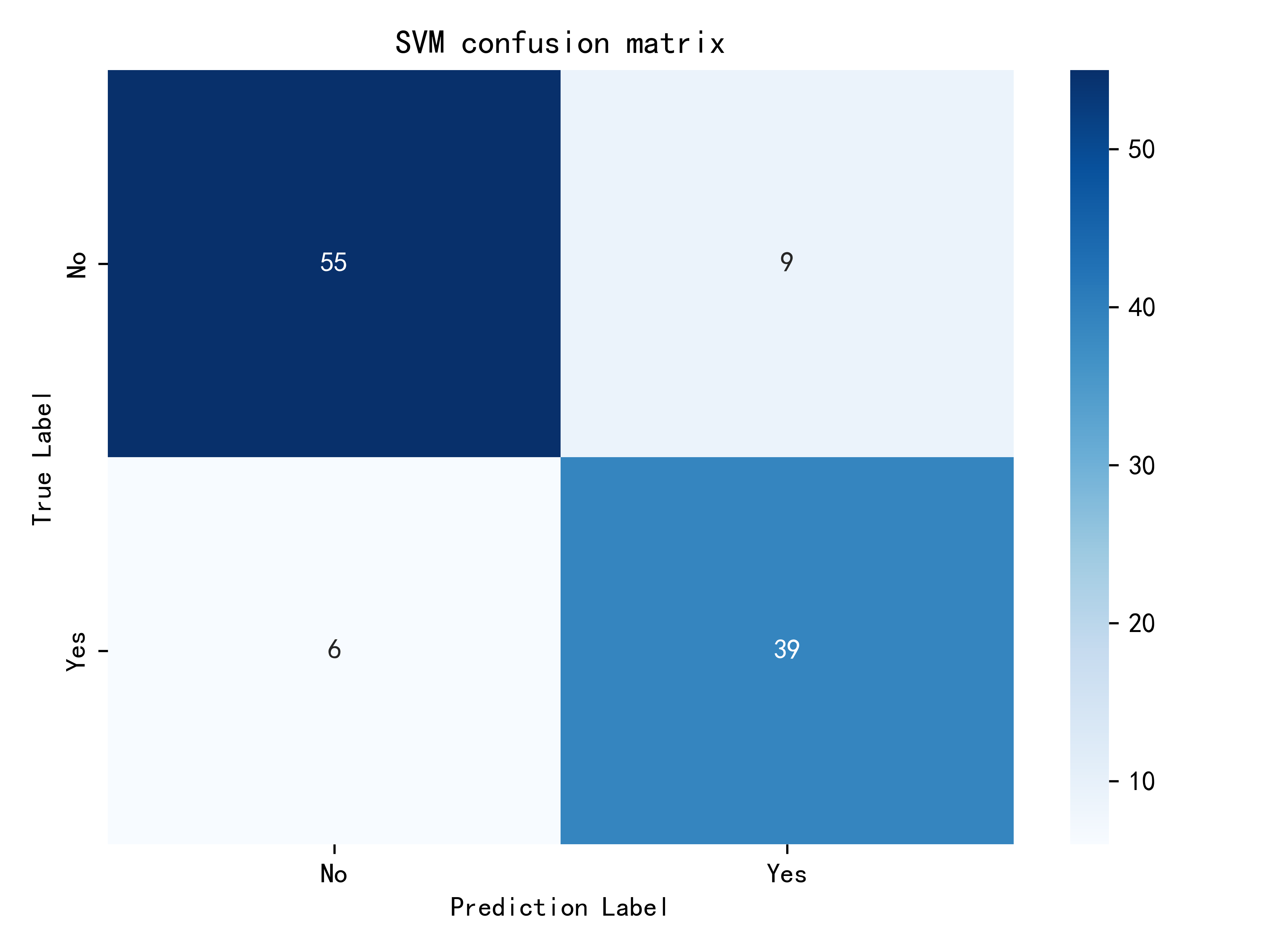

Supplement: Supplementary file 1 [file DataSheet1.zip › Supplementary material/Figure 6.SVM.png]

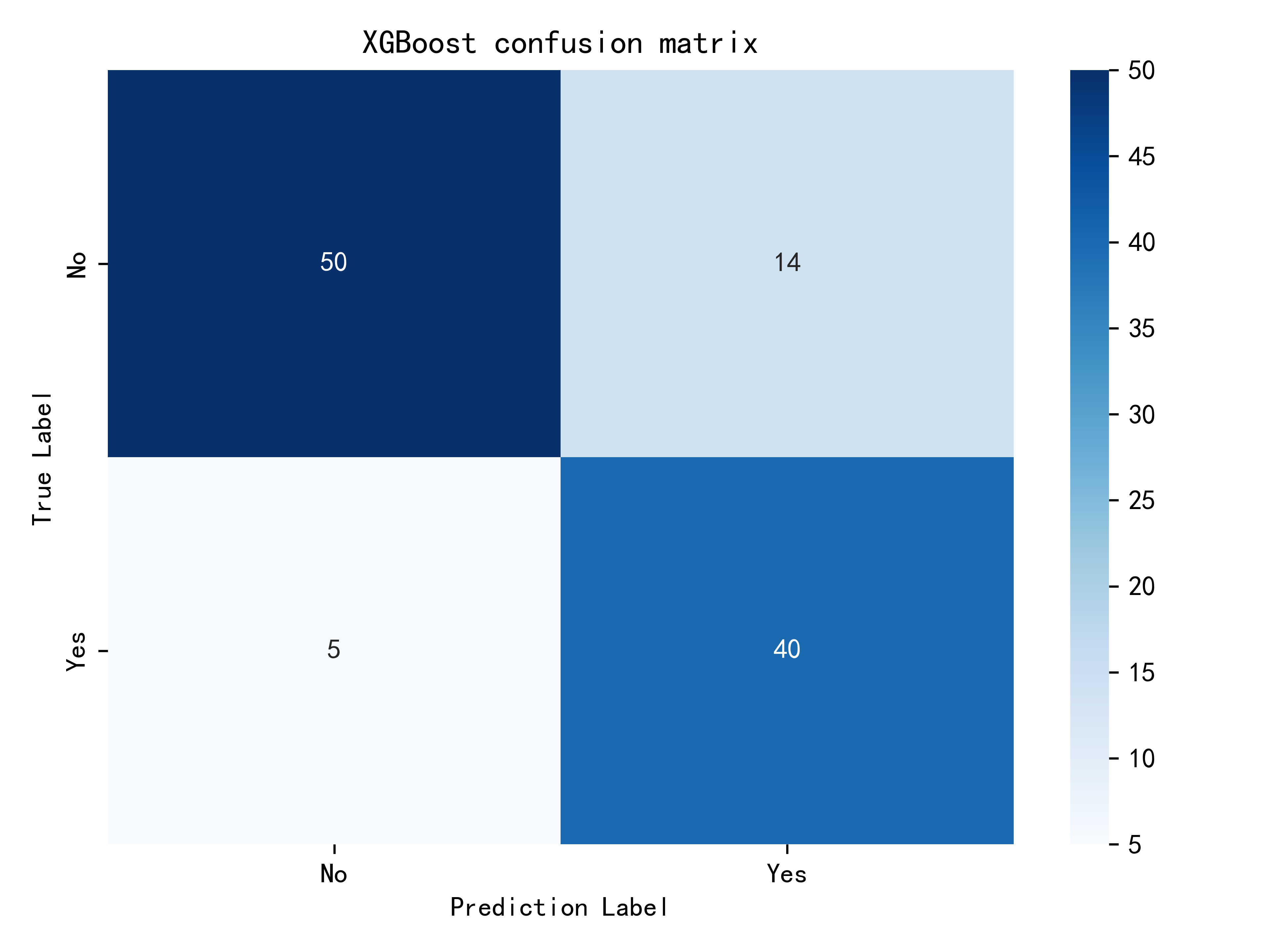

Supplement: Supplementary file 1 [file DataSheet1.zip › Supplementary material/Figure 7.XGBoost.png]

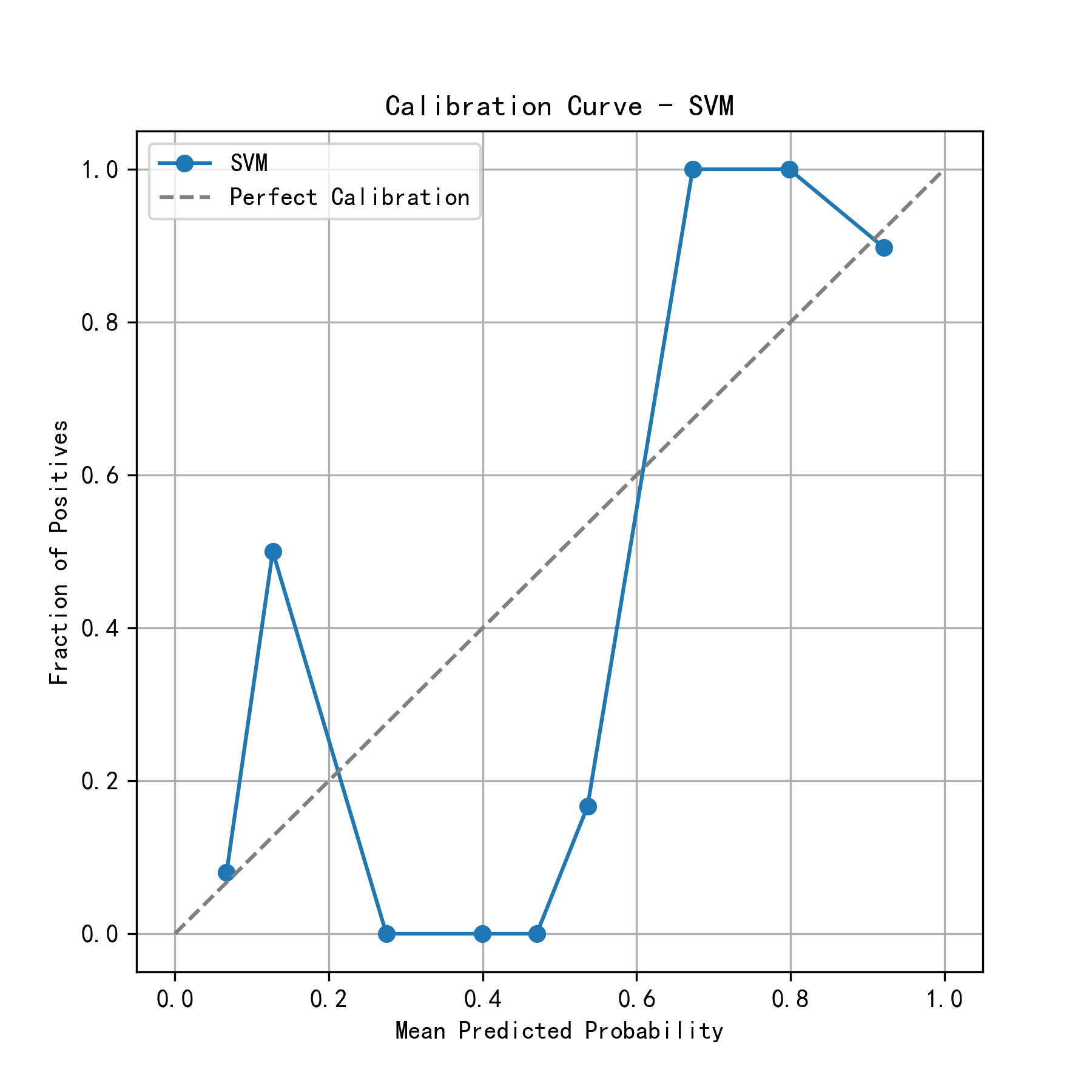

Supplement: Supplementary file 1 [file DataSheet1.zip › Supplementary material/Figure 8 Calibration Curve-SVM .png]

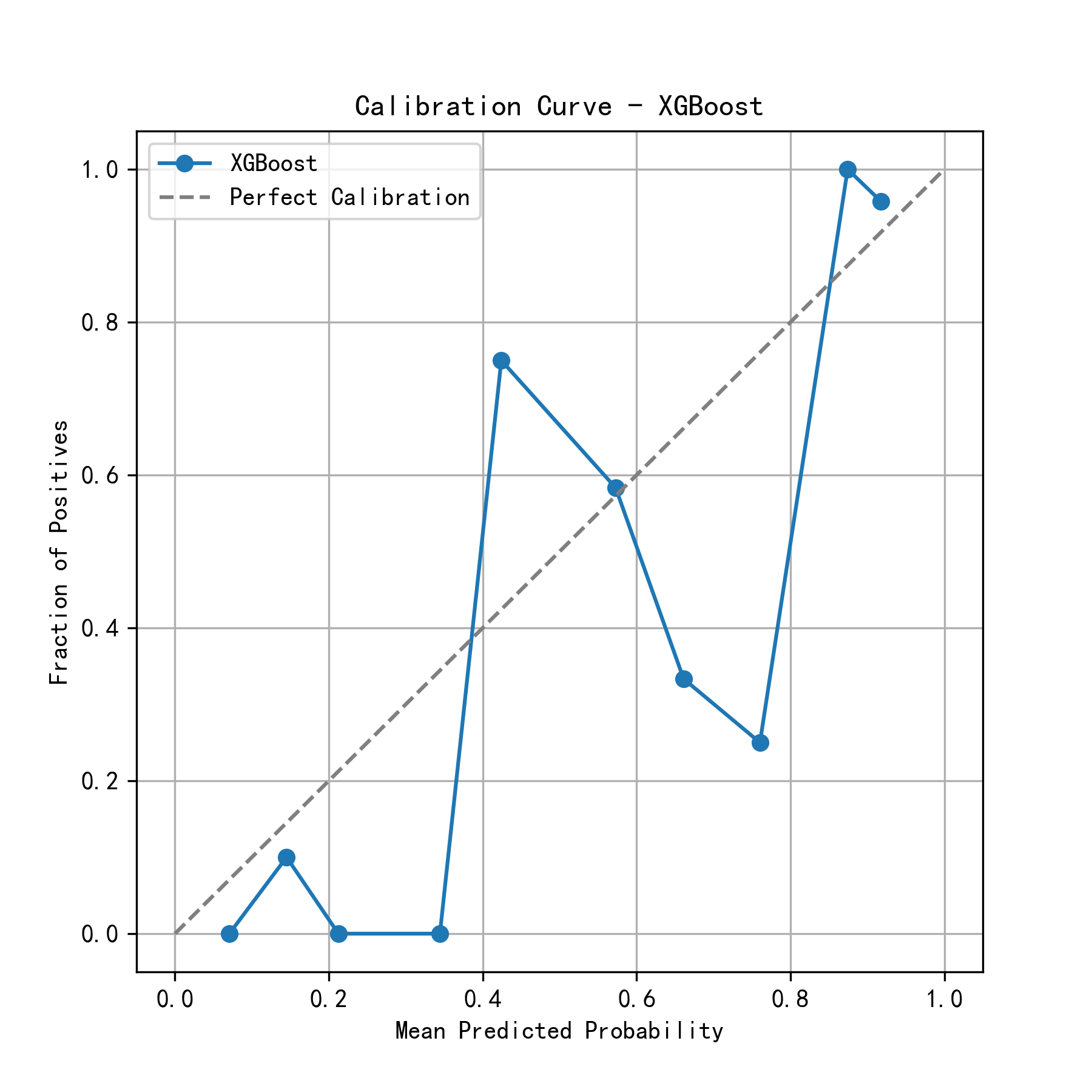

Supplement: Supplementary file 1 [file DataSheet1.zip › Supplementary material/Figure 9. Calibration Curve -XGBoost.png]
